# Impact of COVID-19 In-hospital Mortality in Chagas Disease Patients

**DOI:** 10.3389/fmed.2022.880796

**Published:** 2022-05-09

**Authors:** Gilberto Marcelo Sperandio da Silva, Mauro Felippe Felix Mediano, Michele Ferreira Murgel, Patricia Mello Andrade, Marcelo Teixeira de Holanda, Andréa Rodrigues da Costa, Henrique Horta Veloso, Fernanda Martins Carneiro, Cláudia Maria Valete Rosalino, Andréa Silvestre de Sousa, Fernanda de Souza Nogueira Sardinha Mendes, Roberta Olmo Pinheiro, Valdiléa Gonçalves Veloso, Roberto Magalhães Saraiva, Alejandro Marcel Hasslocher-Moreno

**Affiliations:** ^1^Evandro Chagas National Institute of Infectious Diseases, Fiocruz, Rio de Janeiro, Brazil; ^2^Leprosy Laboratory, Oswaldo Cruz Institute, Fiocruz, Rio de Janeiro, Brazil; ^3^Ibero-American Network for Chagas Disease, Barcelona, Spain

**Keywords:** Chagas disease, SARS-CoV-2, COVID-19, in-hospital mortality, *Trypanosoma cruzi*

## Abstract

The COVID-19 virus infection caused by the new SARS-CoV-2 was first identified in Rio de Janeiro (RJ), Brazil, in March 2020. Until the end of 2021, 504,399 COVID-19 cases were confirmed in RJ, and the total death toll reached 68,347. The Evandro Chagas National Institute of Infectious Diseases from Oswaldo Cruz Foundation (INI-Fiocruz) is a referral center for treatment and research of several infectious diseases, including COVID-19 and Chagas disease (CD). The present study aimed to evaluate the impact of COVID-19 on in-hospital mortality of patients with CD during the COVID-19 pandemic period. This observational, retrospective, longitudinal study evaluated all patients with CD hospitalized at INI-Fiocruz from May 1, 2020, to November 30, 2021. One hundred ten hospitalizations from 81 patients with CD (58% women; 68 ± 11 years) were evaluated. Death was the study's main outcome, which occurred in 20 cases. The mixed-effects logistic regression was performed with the following variables to test whether patients admitted to the hospital with a COVID-19 diagnosis would be more likely to die than those admitted with other diagnoses: admission diagnosis, sex, age, COVID-19 vaccination status, CD clinical classification, and the number of comorbidities. Results from multiple logistic regression analysis showed a higher risk of in-hospital mortality in patients diagnosed with COVID-19 (OR 6.37; 95% CI 1.78–22.86) compared to other causes of admissions. In conclusion, COVID-19 infection had a significant impact on the mortality risk of INI-Fiocruz CD patients, accounting for one-third of deaths overall. COVID-19 presented the highest percentage of death significantly higher than those admitted due to other causes during the COVID-19 pandemic.

## Introduction

Chagas disease (CD) is a neglected tropical disease affecting the cardiovascular, digestive and central nervous systems (1). Chagas disease affects about 6–7 million people worldwide, and in Brazil, more than 1 million people are infected with *Trypanosoma cruzi* (*T. cruzi*) (2, 3). On 31 December 2019, the WHO was informed of many acute respiratory syndrome cases from unknown etiology detected in Wuhan City, Hubei Province of China. Few months later, WHO declared the COVID-19 pandemic caused by a new coronavirus (SARS-COV2) (4). By the end of 2021, the reported cases of COVID-19 in Brazil were 22,287,521, and the total death toll was 619,056 (5). In the same period, the state of Rio de Janeiro registered 504,399 cases and 68,347 deaths due to COVID-19 (6). Considering that Evandro Chagas National Institute of Infectious Diseases of Oswaldo Cruz Foundation (INI-Fiocruz) is a referral infectious disease center institution in Brazil, the Brazilian Ministry of Health supported the construction of a large hospital unit to face the COVID-19 pandemic, with a capacity for up to 200 beds for the intensive and semi-intensive treatment of patients infected by Sars-CoV-2 (7).

The literature regarding *T. cruzi*/SARS-CoV-2 coinfection is still scarce, especially considering patients with Chagas heart disease (CHD) (8). In addition, CHD involves a complex interaction between different processes related to tissue damage due to parasite persistence, inflammation, specific immune response, among other physiological abnormalities (9). Another important aspect to be considered in the case of *T. cruzi*/SARS-CoV-2 coinfection is that the CD population is aging, with individuals with CD also presenting a high prevalence of comorbidities (10, 11). Patients with CHD, especially those with comorbidities, may develop severe forms of COVID-19 (12).

A recent study on mortality in CD and COVID-19 coinfection showed that the presence of comorbidities and their number increased the risk of COVID-19 death (13). In addition, this study identified deaths of patients with CD followed at INI-Fiocruz, through an active search in the Extrajudicial Portal for consultations on births and deaths of the Judiciary of the state of Rio de Janeiro, who died during the pandemic period. It was observed that 31.43% of deaths had COVID-19 as the main cause. Molina et al. in a study that compared hospitalization outcomes in patients with CD and without CD, both with COVID-19 in 11 Brazilian hospitals, observed a similar mortality rate (32.3%) (14). Another study that evaluated the profile of deaths in Brazilian hospitals showed that COVID-19 was the main cause of death in 2020, surpassing deaths from cardiovascular diseases (15).

The preliminary evidence raised by these previous studies addressed CD patients with COVID-19, but did not compare hospitalized CD patients with and without COVID-19. Therefore, the present study aimed to assess the impact of COVID-19 on hospital mortality of patients with CD during the COVID-19 pandemic period.

## Materials and Methods

### Patients and Procedures

The present work is a single-center retrospective cohort study based on data collected from medical records of patients admitted at INI-Fiocruz hospital, located in Rio de Janeiro, Brazil, between May 1, 2020, and November 30, 2021.

All adult patients with CD diagnosis, confirmed by two different serological tests (ELISA, indirect immunofluorescence assay, or chemiluminescence test), and who had been admitted to INI-Fiocruz hospital center were included in this study.

The Brazilian consensus on Chagas disease (3) was used to assess the degree of cardiac involvement of the disease, based on electrocardiogram (EKG), echocardiogram (ECHO) and presence of heart failure (HF). Patients were classified into the following stages: indeterminate (normal EKG and ECHO, without HF); A (altered EKG, normal ECHO and no HF); B1 (altered EKG, altered ECHO with ventricular ejection fraction ≥45%, without HF); B2 (altered EKG, altered ECHO with ventricular ejection fraction <45%, without HF), C (altered EKG and ECHO with compensated HF); and D (altered EKG and ECHO with decompensated and treatment-refractory HF). Patients with symptoms such as fever, cough, sore throat, runny nose, and dyspnea were considered suspects of COVID-19. COVID-19 diagnosis was confirmed by SARS-CoV-2 positivity in real-time reverse-transcription PCR (RT-PCR) (Biomanguinhos kit [E+P1], Fiocruz, Brazil) of nasopharyngeal secretion and/or bronchoalveolar fluid. Patients with negative RT-PCR, but who had an epidemiological history and/or symptoms and clinical signs compatible with COVID-19, computed tomography (CT) chest with changes characteristic of COVID-19 pneumonia, including the presence of ground-glass opacities, was considered for the diagnosis.

The variables considered were age, sex, education level, COVID-19 vaccine, cause of hospital admission (COVID-19 vs. other causes), clinical features (oxygen saturation [SpO2], dyspnea, nausea, vomiting, cough, and fever), the need for respiratory support, RT-PCR results for SARS-COV-2 virus, hospitalization time, CD clinical forms (indeterminate, cardiac or digestive), and presence of comorbidities (systemic arterial hypertension, diabetes mellitus, dyslipidemia, and asthma).

Research Electronic Data Capture (REDCap) and Software R version 4.0.4 were used for data entry and analysis, respectively. Statistical analyzes were performed using the RStudio with the packages epiDisplay: Epidemiological Data Display Package (16). A statistical analysis was performed by descriptive analysis with numerical variables expressed as the mean + SD and the median. Student's *t*-test or the Mann–Whitney U-test was used to compare means between pairs of groups. Pearson's x2 test or Fisher's exact test for categorical variables was used for simple analysis of each variable for hospital admission (COVID-19 vs. other causes). Multivariate mixed logistic models were performed to test the hypothesis of whether patients admitted to the hospital with a COVID-19 diagnosis would be more likely to die than those admitted with other diagnoses. The mixed-effects logistic regression was performed using the glmer function in the lme4 R package, and estimates of the odds ratios were calculated using the tidy function in the broom mixed package. Models included a random intercept by patient. Multivariate analyses included the cause of hospital admission as the exposure variable (COVID-19 vs. other causes). They were adjusted by potential confounders such as sex, age, COVID-19 vaccination status, CD classification, and the number of comorbidities. Statistical significance was set at *p*-value < 0.05.

## Results

A total of 81 CD patients were included; most of them were women, elderly individuals with <9 years of schooling. Most patients presented the CD cardiac form. There was a high prevalence of comorbidities, mainly systemic arterial hypertension, diabetes mellitus, and dyslipidemia. Only one-fifth of the patients reported no comorbidities (Table 1).

**Table 1:** Demographics and clinical characteristics of CD patients admitted to the INI-Fiocruz hospital (*n* = 81).

**Variable**	**n (%)**
**Included patients**	**81 (100%)**
**Sex**	
- men	34 (42)
- women	47 (58)
**Age**	
- mean(SD)	67.7 (10.7)
**Level of Education**	
≥ 9 years	8 (9.9)
<9 years	63 (77.8)
Illiterate	10 (12.3)
**CD clinical forms**	
Indeterminate Form	13 (16)
A	19 (23.5)
B1	12 (14.8)
B2	7 (8.6)
C	28 (34.6)
D	2 (2.5)
CD cardiac form (A+B1+B2+C+D)	68 (84)
**Systemic Arterial Hypertension**	
NO	36 (44.4)
YES	45 (55.6)
**Diabetes Mellitus**	
NO	51 (63)
YES	30 (37)
**Dyslipidemia**	
NO	35 (43.2)
YES	46 (56.8)
**Number of comorbidities**	
None	18 (22.2)
One	23 (28.4)
Two	18 (22.2)
Three or more	22 (27.2)

A total of 110 hospital admissions were observed in this study. Of the 81 patients, 61 patients were admitted once, 14 patients were admitted twice, three patients were admitted three times, and three patients were admitted four times during the study period. The frequency of sex, mean age, frequency of level of education, CD clinical forms, RT-PCR test, mean O2 saturation, frequency of COVID-19 vaccination, fever, cough, dyspnea, anosmia, CT chest exam, mean of hospitalization time, intensive care unit (ICU) admission, frequency of systemic arterial hypertension, diabetes mellitus, dyslipidemia, asthma, and number of comorbidities are depicted in Table 2. Regarding hospitalizations due to COVID-19, there was a significative higher frequency of positive PCR for COVID-19 (81.2%), fever (58.8%), cough (64.7%), dyspnea (82.4%), anosmia (11.8%), TC chest with presence of ground-glass opacities (82.4%), and admission on ICU (82.4%) than in patients admitted due to other causes (0%, 6.5%, 2.2%, 22.6%, 0%, 9.7% and 26.9%, respectively; all *p* < 0.05). Regarding the primary outcome of this study, there was a significative higher frequency of death in patients admitted due to COVID-19 (41.2%, *p* < 0.001) than in others (14%) (Table 2).

**Table 2:** Comparison of demographic and clinical characteristics between hospital admissions due to COVID-19 and other causes (*n* = 110).

	**Hospital admission**		
	**n (%)** ^*^		
**Variable**	**No COVID-19 *n* = 93 (%)**	**COVID-19 *n* = 17 (%)**	***p*-value**
**Sex**			0.914
Male	37 (39.8)	7 (41.2)	
Female	56 (60.2)	10 (58.8)	
**Age**			0.557
Median (IQR)	69.6 (62.6, 76)	72.1 (61.9, 76.3)	
**Level of Education**			0.253
≥ 9 years	6 (6.5)	3 (17.6)	
<9 years	77 (82.8)	13 (76.5)	
Illiterate	10 (10.8)	1 (5.9)	
**CD clinical forms**			0.942
Indeterminate Form	15 (16.1)	2 (11.8)	
A	19 (20.4)	5 (29.4)	
B1	12 (12.9)	2 (11.8)	
B2	11 (11.8)	1 (5.9)	
C	34 (36.6)	7 (41.2)	
D	2 (2.2)	0 (0)	
Total CD cardiac form (A + B1 + B2 + C + D)	78 (83.9)	15 (88.2)	
**RT-PCR**			<0.001
Negative	31 (100)	3 (18.8)	
Positive	0 (0)	13 (81.2)	
**O2 Saturation**			0.313
Mean (SD)	97.1 (1.7)	96.2 (2.6)	
**COVID-19 Vaccination**			0.55
No	63 (71.6)	14 (82.4)	
Yes	25 (28.4)	3 (17.6)	
**Fever**			<0.001
No	87 (93.5)	7 (41.2)	
Yes	6 (6.5)	10 (58.8)	
**Cough**			<0.001
No	91 (97.8)	6 (35.3)	
Yes	2 (2.2)	11 (64.7)	
**Dyspnea**			<0.001
No	72 (77.4)	3 (17.6)	
Yes	21 (22.6)	14 (82.4)	
**Nausea/Vomiting**			1
No	78 (83.9)	15 (88.2)	
Yes	15 (16.1)	2 (11.8)	
**Anosmia**			0.023
No	93 (100)	15 (88.2)	
Yes	0 (0)	2 (11.8)	
**CT Chest—Ground-Glass opacities**			
Presence	9 (9.7)	14 (82.4)	<0.001
Normal results	84 (90.3)	3 (17.6)	
**Hospitalization time**			0.362
Median (IQR)	7 (4, 13)	7 (6, 18)	
**Intensive Care Unit admission**			<0.001
No	68 (73.1)	3 (17.6)	
Yes	25 (26.9)	14 (82.4)	
**Systemic Arterial Hypertension**			0.885
No	42 (45.2)	8 (47.1)	
Yes	51 (54.8)	9 (52.9)	
**Diabetes Mellitus**			0.419
No	64 (68.8)	10 (58.8)	
Yes	29 (31.2)	7 (41.2)	
**Dyslipidemia**			0.404
No	43 (46.2)	6 (35.3)	
Yes	50 (53.8)	11 (64.7)	
**Asthma**			0.023
No	93 (100)	15 (88.2)	
Yes	0 (0)	2 (11.8)	
**Number of comorbidities**			0.734
None	23 (24.7)	3 (17.6)	
One	28 (30.1)	4 (23.5)	
Two	20 (21.5)	6 (35.3)	
**Three or more**	22 (23.7)	4 (23.5)	
Median (IQR)	1 (1, 2)	2 (1, 2)	
**Patient's outcome**			<0.001
Death due to COVID-19	0 (0)	7 (41.2)	
Death due Other	13 (14)	0 (0)	
Hospital discharge	80 (86)	10 (58.8)	

*The number of hospital admissions was higher than the number of patients because 20 patients were admitted twice or more.

The multiple logistic regression analysis considering the 110 hospitalizations showed a higher risk of in-hospital mortality in patients diagnosed with COVID-19 after adjustments for potential confounders (Table 3).

**Table 3:** Univariate and Multivariate Logistic regression results to assess whether CD patients diagnosed with COVID-19 on hospital admission have a higher chance of in-hospital mortality.

	**Univariate Logistic regression**	**Multivariate Logistic regression**
**Variable**	**OR**^**C**^ **(95% CI;** ***P*** **value)**	**OR**^**A**^ **(95% CI;** ***P*** **value)**
COVID-19 hospitalization vs. other causes	4.31 (1.39–13.33; 0.01)	6.37 (1.78–22.86; 0.004)

^C^*Univariate regression results and ^A^multivariate-adjusted regression results*.

## Discussion

Little is known about the impact of COVID-19 on the mortality of hospitalized CD patients during the COVID-19 pandemic. This study analyzed patients with CD who were admitted to a Brazilian hospital center specialized in the treatment of infectious diseases.

The mean age, sex, and comorbidities frequency presented by the patients of the present study were similar to those previously described in ambulatorial patients in our cohort (17). However, the prevalence of CHD patients in this study was higher (84% against 53.6%) than those previously described. This is expected since patients with the cardiac form are more likely to be hospitalized than those who do not have heart disease. In addition, previous studies suggest that systemic arterial hypertension, diabetes mellitus, and prior cardiovascular disease are important risk factors for severity and mortality in COVID-19 infected people (12).

The aging trend of our cohort and a higher prevalence of women had also been previously reported (10). Other study corroborates the previous aging trend and suggests an increase in the average age of the most severe cases (17). Similar to Molina et al. (14), there was a higher frequency of women in the present study and higher mean age among CD patients admitted to the hospital due to COVID-19.

According to Struyf et al. (18), symptoms such as cough, sore throat, fever, myalgia or arthralgia, fatigue, and headache may be present in patients with COVID-19, dyspnea, and anosmia are very specific signs of COVID-19. The present study corroborates that these symptoms are frequent among CD patients with COVID-19.

In our study, the sensitivity of RT-PCR was 81.2%, as described in other studies (19). The three patients diagnosed with COVID-19, and who had negative RT-PCR, had CT chest compatible with COVID-19 pneumonia. Overall, RT-PCR shows low sensitivity in diagnosing patients with COVID-19 infection compared to the CT chest (20).

Molina et al. (14) showed a 32.3 % in-hospital mortality rate in CD patients with COVID-19, similar to the one we described in the present study (33.3%). Moreover, in our study, multivariate analysis indicated that CD patients hospitalized due to COVID-19 had 6 times greater risk of in-hospital mortality than those hospitalized due to other causes. Considering this context, Zimmermann et al. (15) results corroborate our observation, indicating that COVID-19 was the leading cause of death in public hospitals in Brazil in 2020, superior to mortality due to other causes.

In conclusion, COVID-19 infection caused a significant impact on the mortality risk of CD patients. COVID-19 presented the highest percentage of death that was significantly higher than those admitted due to other causes during the COVID-19 pandemic period.

## Data Availability Statement

The raw data supporting the conclusions of this article will be made available by the authors, without undue reservation.

## Ethics Statement

This study was approved by the INI-Fiocruz Research Ethics Committee (number CAAE: 35748820.1.0000.5262) on September 2, 2020, and was carried out in accordance with the 1964 Declaration of Helsinki and its later amendments. Written informed consent for participation was not required for this study in accordance with the national legislation and the institutional requirements.

## Author Contributions

AH-M, MMu, MMe, AC, FC, and PA collected, interpreted data, and prepared tables. GS, AH-M, RS, and MMe analyzed the data. MMe, MMu, PA, MH, AC, HV, FC, CV, AS, FM, RP, VV, RS, and AH-M reviewed and edited the manuscript. GS, MH, CV, AS, FM, RP, VV, AC, HV, FC, MMe, RS, and AH-M conceptualized, designed study, supervised the study, and interpreted data. GS wrote the manuscript. All authors contributed to the article and approved the submitted version.

## Funding

This study was supported by the Evandro Chagas National Institute of Infectious Diseases, Oswaldo Cruz Foundation (Fiocruz), Coordenação de Aperfeiçoamento de Pessoal de Nível Superior (CAPES – Telemedicina e Análise de Dados Médicos no 12/2020, processo: 23038.004292/2020-80), Fundação Carlos Chagas Filho de Amparo à Pesquisa do Estado do Rio de Janeiro (FAPERJ) and Conselho Nacional de Desenvolvimento Científico e Tecnológico (CNPq - bolsa de produtividade, number: 313327/2018_1).

## Conflict of Interest

The authors declare that the research was conducted in the absence of any commercial or financial relationships that could be construed as a potential conflict of interest.

## Publisher's Note

All claims expressed in this article are solely those of the authors and do not necessarily represent those of their affiliated organizations, or those of the publisher, the editors and the reviewers. Any product that may be evaluated in this article, or claim that may be made by its manufacturer, is not guaranteed or endorsed by the publisher.
