# Corrigendum: Impact of COVID-19 in-hospital mortality in chagas disease patients

**DOI:** 10.3389/fmed.2022.963805

**Published:** 2022-07-26

**Authors:** Gilberto Marcelo Sperandio da Silva, Mauro Felippe Felix Mediano, Michele Ferreira Murgel, Patricia Mello Andrade, Marcelo Teixeira de Holanda, Andréa Rodrigues da Costa, Henrique Horta Veloso, Fernanda Martins Carneiro, Cláudia Maria Valete Rosalino, Andréa Silvestre de Sousa, Fernanda de Souza Nogueira Sardinha Mendes, Roberta Olmo Pinheiro, Valdiléa Gonçalves Veloso, Roberto Magalhães Saraiva, Alejandro Marcel Hasslocher-Moreno

**Affiliations:** ^1^Evandro Chagas National Institute of Infectious Diseases, Fiocruz, Rio de Janeiro, Brazil; ^2^Leprosy Laboratory, Oswaldo Cruz Institute, Fiocruz, Rio de Janeiro, Brazil; ^3^Ibero-American Network for Chagas Disease, Barcelona, Spain

**Keywords:** Chagas disease, SARS-CoV-2, COVID-19, in-hospital mortality, *Trypanosoma cruzi*

**Addition of an Author**

In the published article, there was an error in the author list, and authors **A**ndréa Rodrigues da Costa, Henrique Horta Veloso, and Fernanda Martins Carneiro were erroneously excluded. The corrected author list appears below.

Gilberto Marcelo Sperandio da Silva^1*^, Mauro Felippe Felix Mediano^1^, Michele Ferreira Murgel^1^, Patricia Mello Andrade^1^, Marcelo Teixeira de Holanda^1^, Andréa Rodrigues da Costa^1^, Henrique Horta Veloso^1^, Fernanda Martins Carneiro^1^, Cláudia Maria Valete Rosalino^1^, Andréa Silvestre de Sousa^1^, Fernanda de Souza Nogueira Sardinha Mendes^1^, Roberta Olmo Pinheiro^2^, Valdiléa Gonçalves Veloso^1^, Roberto Magalhães Saraiva^1, 3^ and Alejandro Marcel Hasslocher-Moreno^1, 3^

The corrected Author Contributions Statement appears below.

AH-M, MMu, MMe, AC, FC, and PA collected, interpreted data, and prepared tables. GS, AH-M, RS, and MMe analyzed the data. MMe, MMu, PA, MH, AC, HV, FC, CV, AS, FM, RP, VV, RS, and AH-M reviewed and edited the manuscript. GS, MH, CV, AS, FM, RP, VV, AC, HV, FC, MMe, RS, and AH-M conceptualized, designed study, supervised the study, and interpreted data. GS wrote the manuscript. All authors contributed to the article and approved the submitted version.

The authors apologize for this inclusion and state that this does not change the scientific conclusions of the article in any way. The original article has been updated.

## Publisher's note

All claims expressed in this article are solely those of the authors and do not necessarily represent those of their affiliated organizations, or those of the publisher, the editors and the reviewers. Any product that may be evaluated in this article, or claim that may be made by its manufacturer, is not guaranteed or endorsed by the publisher.

